# Interleukin-21 sustains inflammatory signals that contribute to sporadic colon tumorigenesis

**DOI:** 10.18632/oncotarget.3532

**Published:** 2015-03-12

**Authors:** Veronica De Simone, Giulia Ronchetti, Eleonora Franzè, Alfredo Colantoni, Angela Ortenzi, Massimo C. Fantini, Angelamaria Rizzo, Giuseppe S. Sica, Pierpaolo Sileri, Piero Rossi, Thomas T. MacDonald, Francesco Pallone, Giovanni Monteleone, Carmine Stolfi

**Affiliations:** ^1^ Department of Systems Medicine, University of Rome “Tor Vergata”, Rome, Italy; ^2^ Department of Surgery, University of Rome “Tor Vergata”, Rome, Italy; ^3^ Centre for Immunology and Infectious Disease, Blizard Institute of Cell and Molecular Science, Barts and the London School of Medicine and Dentistry, London, UK

**Keywords:** *Apc*^min/+^ mice, STAT3, COX-2/PGE2, VEGF

## Abstract

Interleukin (IL)-21 triggers inflammatory signals that contribute to the growth of neoplastic cells in mouse models of colitis-associated colorectal cancer (CRC). Because most CRCs are sporadic and arise in the absence of overt inflammation we have investigated the role of IL-21 in these tumors in mouse and man. IL-21 was highly expressed in human sporadic CRC and produced mostly by IFN-γ-expressing T-bet/RORγt double-positive CD3+CD8− cells. Stimulation of human CRC cell lines with IL-21 did not directly activate the oncogenic transcription factors STAT3 and NF-kB and did not affect CRC cell proliferation and survival. In contrast, IL-21 modulated the production of protumorigenic factors by human tumor infiltrating T cells. IL-21 was upregulated in the neoplastic areas, as compared with non-tumor mucosa, of *Apc*^min/+^ mice, and genetic ablation of IL-21 in such mice resulted in a marked decrease of both tumor incidence and size. IL-21 deficiency was associated with reduced STAT3/NF-kB activation in both immune cells and neoplastic cells, diminished synthesis of protumorigenic cytokines (that is, IL-17A, IL-22, TNF-α and IL-6), downregulation of COX-2/PGE2 pathway and decreased angiogenesis in the lesions of *Apc*^min/+^ mice. Altogether, data suggest that IL-21 promotes a protumorigenic inflammatory circuit that ultimately sustains the development of sporadic CRC.

## INTRODUCTION

Accumulation of mutations in various oncogenes and tumor suppressor genes drives the development of sporadic CRC [1], but at the same time progression of this neoplasia is tightly controlled by complex interactions between cancer cells and immune cells in the tumor microenvironment, with the latter modulating both pro- and anti-tumorigenic pathways [2]. Although these phenomena have been widely documented in CRC arising in patients with inflammatory bowel disease and in mouse models of chemically induced colitis-associated CRC [3-9], recent studies have clearly shown that an immune/inflammatory infiltrate is also present in sporadic CRC, and immune cell-derived cytokines sustain CRC cell growth and spread [10]. It is however possible that distinct immune mechanisms contribute to the pathogenesis of colitis-associated CRC in mouse and man compared to sporadic CRC.

The pleiotropic cytokine interleukin (IL)-21 has potent anti-tumor effects due to its ability to expand the pool of cytotoxic CD8+ T cells, NK cells and NKT cells [11] and beneficial IL-21-mediated anti-tumor responses have been documented in mice implanted with syngeneic tumor lines (including CRC cell lines) [12] as well as in patients with advanced solid tumors [13, 14]. On the other hand, IL-21 also drives Th17 immune responses [15, 16], which promote colon carcinogenesis in mice and negatively influence the prognosis of patients with sporadic CRC [10, 17, 18]. Recent studies from our laboratory have shown that IL-21 is highly expressed in the mucosa of patients with ulcerative colitis complicated by CRC [9], and also plays a key role in sustaining the growth of cancer cells in mice with colitis-associated CRC [9]. Therefore, there is the possibility that IL-21 could have opposing functions on the growth of CRCs, depending on the neoplastic triggers and local immune activation. Since our studies have also shown high IL-21 production in the tumor areas of patients with sporadic CRC [9], we hypothesized that this cytokine can sustain the growth of sporadic CRC.

## RESULTS

### IFN-γ-expressing T-bet/RORγt double-positive CD3+CD8− cells are a major source of IL-21 in human sporadic CRC

**Figure 1:**
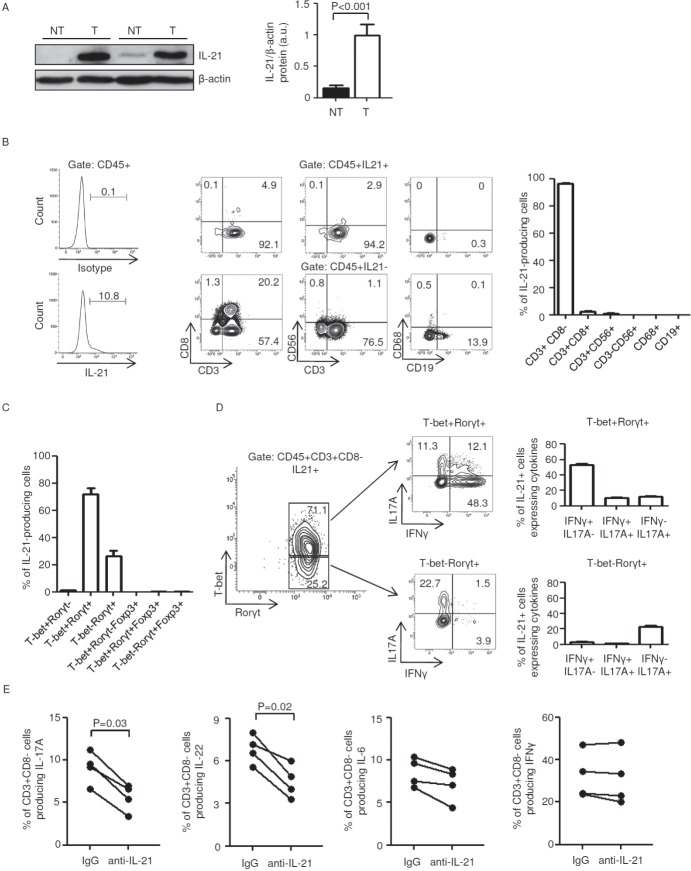
Characterization of IL-21-producing cells in human sporadic colorectal cancer (CRC) A. Total proteins extracted from both tumor (T) and non-tumor (NT) areas of two patients with sporadic CRC were evaluated for IL-21 expression by western blotting. β-actin was used as loading control. The figure is representative of five separate experiments. Right inset. Quantitative analysis of IL-21/β-actin protein ratio. Values are expressed in arbitrary units (a.u.) and indicate the mean ± SEM of all experiments (n=10). B. IL-21 is preferentially made by CD3+CD8− cells in tumor infiltrating leukocytes (TILs). Left panel. Representative histograms showing the percentage of CD45+IL-21+ cells in TILs. The example is representative of five experiments in which cells isolated from five patients with sporadic CRC were analyzed. Numbers above lines indicate the percentages of CD45+IL-21+ cells. Staining of cells with PE-conjugated control isotype IgG is also shown. Central panels. Representative dot-plots showing the fraction of IL-21+ cells in TILs. CD45+IL-21+ and CD45+IL-21- cells were gated and then analyzed for the indicated markers. The numbers indicate the percentage of cells in the designated quadrants. The example is representative of five experiments in which cells isolated from five patients with sporadic CRC were analyzed. Right panel. Representative histograms showing the fraction of IL-21+ cells in TILs. CD45+IL-21+ cells were gated and then analyzed for the indicated markers. Data are expressed as mean ± SEM of five experiments in which cells isolated from five patients with sporadic CRC were analyzed. C-D. IL-21 is preferentially made by IFN-γ-expressing T-bet/RORγt double-positive CD3+CD8− cells in human sporadic CRC. C. Representative histograms showing the percentages of IL-21-producing CD3+CD8− cells expressing the indicated transcription factors. Data are expressed as mean ± SEM of five experiments in which TILs isolated from five patients with sporadic CRC were analyzed. D. IL-17A and IFN-γ expression was analyzed in T-bet-RORγt+ and T-bet/RORγt double-positive IL-21-producing CD3+CD8− cells by flow cytometry. The numbers indicate the percentage of cells in the designated quadrants. Right panels. Percentages of T-bet-RORγt+ and T-bet/RORγt double-positive IL-21-producing CD3+CD8− cells co-expressing IL-17A and/or IFN-γ. Data are expressed as mean ± SEM of five experiments in which TILs isolated from five patients with sporadic CRC were analyzed. E. TILs isolated from four patients with sporadic CRC were cultured either with a control IgG or anti-IL-21 antibody (both used at 10 μg/ml) for 48 h. Each point represents the percentage of IL-17A-, IL-22-, IL-6- and IFN-γ-producing CD45+CD3+CD8− cells, assessed by flow cytometry, in TILs isolated from a single patient.

IL-21 was significantly increased in CRC samples as compared to non-tumor mucosa (Figure 1A). To assess the cellular source of IL-21 in human sporadic CRC, we isolated tumor infiltrating leukocytes (TILs) from patients who underwent surgical resection. Flow cytometry analysis revealed that IL-21 was mostly produced by CD3+CD8− T cells (Figure 1B) and minimally expressed by CD3+CD8+ T cells and natural killer (NK) T cells (CD3+CD56+ cells) (2.54±0.4 and 0.96±0.28 respectively); no IL-21 staining was seen in NK cells (CD3-CD56+ cells), macrophages (CD68+ cells) and B cells (CD19+ cells) thus ruling out such cells as sources of IL-21 in human CRC (Figure 1B). Next, we evaluated whether IL-21 was co-expressed with Th1/Th17 signature cytokines (that is, IFN-γ and IL-17A respectively) within CD3+CD8− and CD3+CD8+ populations. Nearly 5% of CD3+CD8− cells produced IL-21 either alone or in combination with IFN-γ (5.15±0.66 and 4.45±0.67 respectively), whereas less then 2% (1.65±0.46) of such cells co-expressed IL-21 and IL-17A (Suppl. Figure 1A and B). Most of the CD3+CD8+ cells synthesized IFN-γ (77.1±8.25), whereas less than 3% of such cells expressed either IL-21 alone (0.3±0.11) or in combination with IFN-γ or IL-17A (2.8±1.17 and 0.42±0.14 respectively) (Suppl. Figure 1C and D).

The majority of IL-21-producing CD3+CD8− cells were T-bet/receptor-related orphan receptor gamma t (RORγt) double-positive and, to a lesser extent, T-bet-RORγt+ (Figure 1C). Notably, co-expression of the regulatory T cell-associated transcription factor Forkhead box P3 (FoxP3) in T-bet/RORγt double-positive and T-bet-RORγt+ CD3+CD8−cells associated with IL-17A but not IL-21 production (Figure 1C and Suppl. Figure 2). Nearly 50% of IL-21-producing T-bet/RORγt double-positive CD3+CD8− cells co-expressed IFN-γ alone, whereas less than 15% of such cells co-expressed either IL-17A alone or IL-17A and IFN-γ (Figure 1D). One fifth of IL-21-producing T-bet-RORγt+ CD3+CD8− cells co-expressed IL-17A alone, whereas less than 3% of such cells co-expressed either IFN-γ alone or IL-17A and IFN-γ (Figure 1D).

These findings indicate that IL-21 is preferentially made by IFN-γ-expressing T-bet/RORγt double-positive CD3+CD8− cells in human sporadic CRC.

### IL-21 does not directly affect STAT3/NF-kB activation, proliferation and survival in human CRC cells

The transcription factors STAT3 and NF-kB are key mediators in the interplay between immune/inflammatory cells and malignant cells and their activation stimulates proliferation and survival of CRC cells [19]. IL-21 receptor was expressed by DLD-1 and HT-29, two human CRC cell lines (Suppl. Figure 3A). However, no significant change in STAT3 and NF-kB activation was seen in DLD-1 and HT-29 cells following stimulation with increasing doses of IL-21 for different time points (Suppl. Figure 3B and not shown). Similarly, IL-21 did not change the rate of proliferation and the survival of DLD-1 and HT-29 cells, whereas a robust proliferation of both CRC cell lines was seen in the presence of TIL-derived supernatants (Suppl. Figure 3C and D). IL-21 activated STAT3 in human normal lamina propria mononuclear cells (LPMCs) isolated from the macroscopically unaffected colonic mucosa of CRC patients, thus indicating that the cytokine was biologically active (Suppl. Figure 4).

To determine whether IL-21 controls pro- and anti-tumorigenic signals in immune cells infiltrating the tumor, TILs were isolated from sporadic CRC of four patients, activated with anti-CD3/CD28 in the presence of either a control IgG or IL-21 neutralizing antibody and examined by flow cytometry. Blockade of IL-21 reduced the fraction of IL-17A- and IL-22-producing CD3+CD8− cells, whereas no significant changes where observed in the fraction of IL-6- and IFN-γ-producing CD3+CD8− cells (Figure 1E).

### IL-21-knockout-*Apc*^min/+^ mice are resistant to colon tumorigenesis

**Figure 2:**
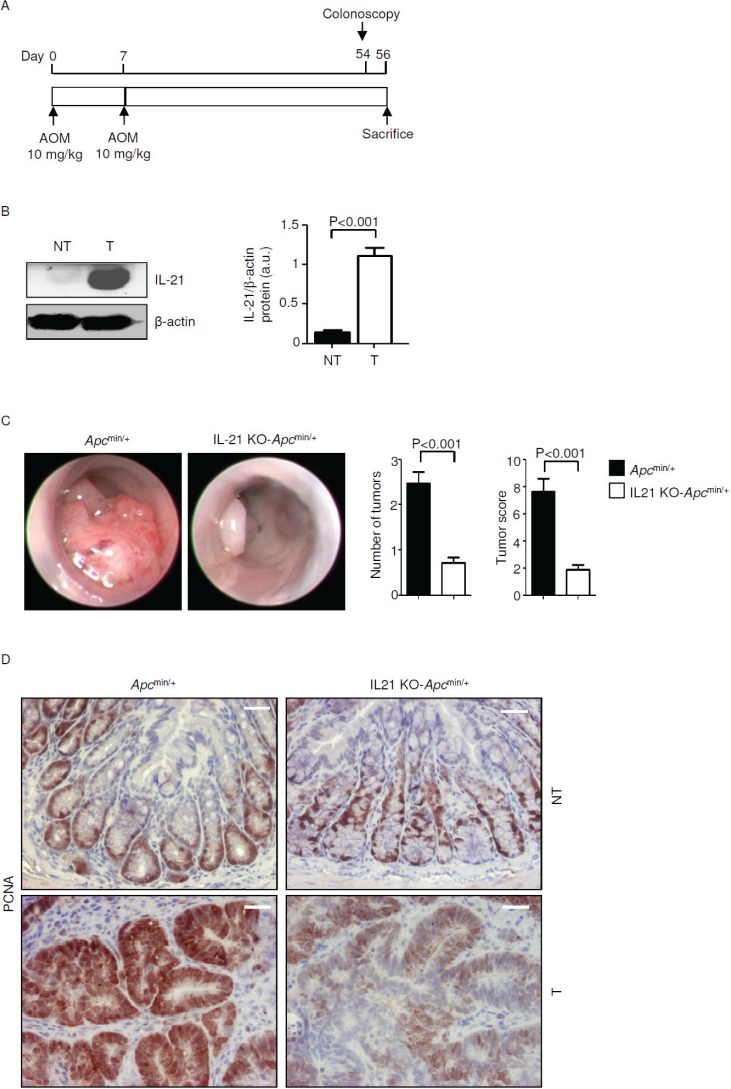
IL-21 deficiency significantly reduces colon tumorigenesis in *Apc*^min/+^ mice A. Experimental protocol used to assess the effect of IL-21 ablation on colon tumorigenesis in *Apc*^min/+^ mice. B. Representative western blotting showing IL-21 expression in colon tissues taken from *Apc*^min/+^ mice killed on day 56. β-actin was used as loading control. One of five representative experiments is shown. NT, non-tumor area, T, tumor area. Right inset. Quantitative analysis of IL-21/β-actin protein ratio in total extracts of T and NT colon tissues taken from *Apc*^min/+^ mice killed on day 56 as measured by densitometry scanning of western blots. Values are expressed in arbitrary units (a.u.) and indicate the mean ± SEM of all experiments (n=5). C. Representative endoscopic pictures of colon tumors developed in *Apc*^min/+^ mice and IL-21 KO-*Apc*^min/+^ mice. Graphs show the number of lesions and the endoscopic scoring of tumors. Data indicate mean ± SEM of four independent experiments in which at least four mice per group were considered. D. Representative images showing PCNA immunostaining in colon sections taken from *Apc*^min/+^ mice and IL-21 KO-*Apc*^min/+^ mice killed on day 56. The scale bars are 20μm. One of four representative experiments in which similar results were obtained is shown.

To further evaluate the role of IL-21 in sporadic CRC *in vivo* we used *Apc*^min/+^ mice. IL-21 was overexpressed in the colonic lesions of *Apc*^min/+^ mice treated with AOM and killed on day 56 (Figure 2A) as compared to the non-tumor areas (Figure 2B). Both *Apc*^min/+^ (control) and IL-21-knockout (IL-21 KO)-*Apc*^min/+^ mice were injected with AOM and monitored for tumor formation. Four *Apc*^min/+^ mice had to be killed before the end of the scheduled treatment, because of intestinal occlusion caused by large colonic tumors, whereas all the IL-21-KO-*Apc*^min/+^ mice survived until the end of the study.

Endoscopy on day 54 showed that control mice developed multiple large tumors whereas the number and size of tumors were reduced in the colon of IL-21 KO-*Apc*^min/+^ mice (Figure 2C). These results were confirmed by direct assessment of tumors in mice killed on day 56 (unpublished data). Proliferating cell nuclear antigen (PCNA) staining confirmed that lack of IL-21 reduces the rate of proliferation in transformed epithelial cells (Figure 2D). By contrast, there was no significant change in PCNA staining in the normal colonic mucosa of control and IL-21-KO-*Apc*^min/+^ mice (Figure 2D).

**Figure 3:**
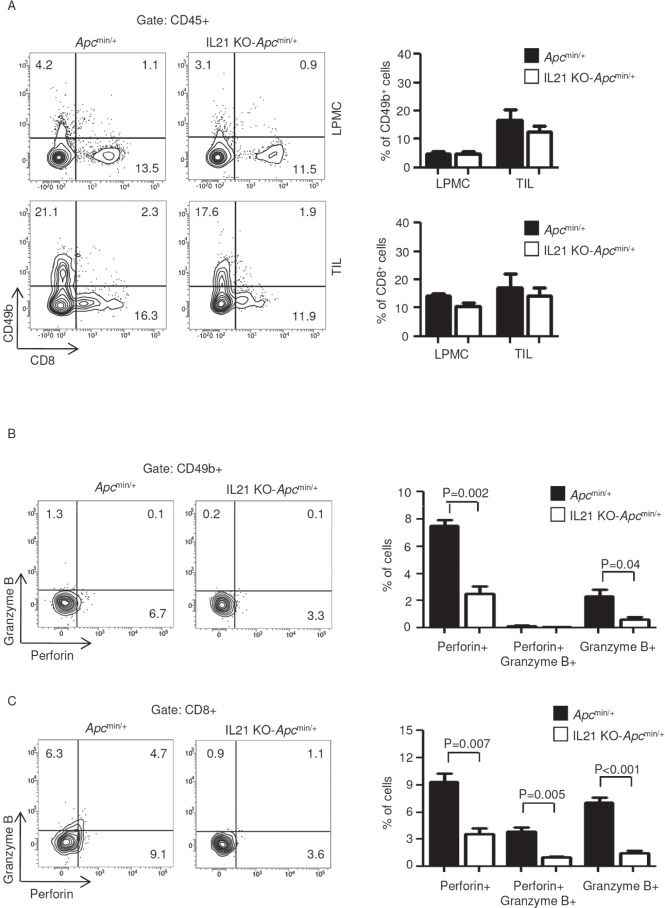
IL-21 deficiency in *Apc*^min/+^ mice associates with a reduced fraction of perforin and/or granzyme B-producing CD49b- and CD8−positive cells in TILs A. Representative dot-plots showing CD49b- and CD8−positive cells in LPMCs and TILs isolated from the colon of *Apc*^min/+^ mice and IL-21 KO-*Apc*^min/+^ mice killed on day 56. Numbers indicate the percentages of cells in the designated quadrants. One of four representative experiments is shown. Right insets. Representative histograms showing the fraction of CD49b- and CD8−positive cells in LPMCs and TILs isolated from the colon of *Apc*^min/+^ mice and IL-21 KO-*Apc*^min/+^ mice killed on day 56. CD45-positive cells were gated and analyzed for the indicated markers. Values are mean ± SEM of two independent experiments containing at least two mice per group. B-C. TILs isolated from the colon of *Apc*^min/+^ mice and IL-21 KO-*Apc*^min/+^ mice killed on day 56 were assessed for perforin- and granzyme B-expressing CD49b- (B) and CD8−positive cells (C) by flow cytometry. The numbers indicate the percentage of cells in the designated quadrants. Right panels. Percentages of CD49b- (B) and CD8−positive cells (C) co-expressing perforin and/or granzyme B. Data are expressed as mean ± SEM of two independent experiments containing at least two mice per group.

To investigate whether IL-21 deficiency affects the fraction and/or the function of immune cell subsets involved in anti-tumor immunity, LPMCs and TILs isolated from *Apc*^min/+^ mice and IL-21 KO-*Apc*^min/+^ mice were analyzed for CD8 and CD49b by flow cytometry. IL-21 deficiency associated with no change in the numbers of cytotoxic T cells and NK cells in both LPMCs and TILs (Figure 3A). However, lack of IL-21 significantly reduced the fraction of perforin- and/or granzyme B-producing NK and CD8 T cells in TILs (Figure 3B and C).

**Figure 4:**
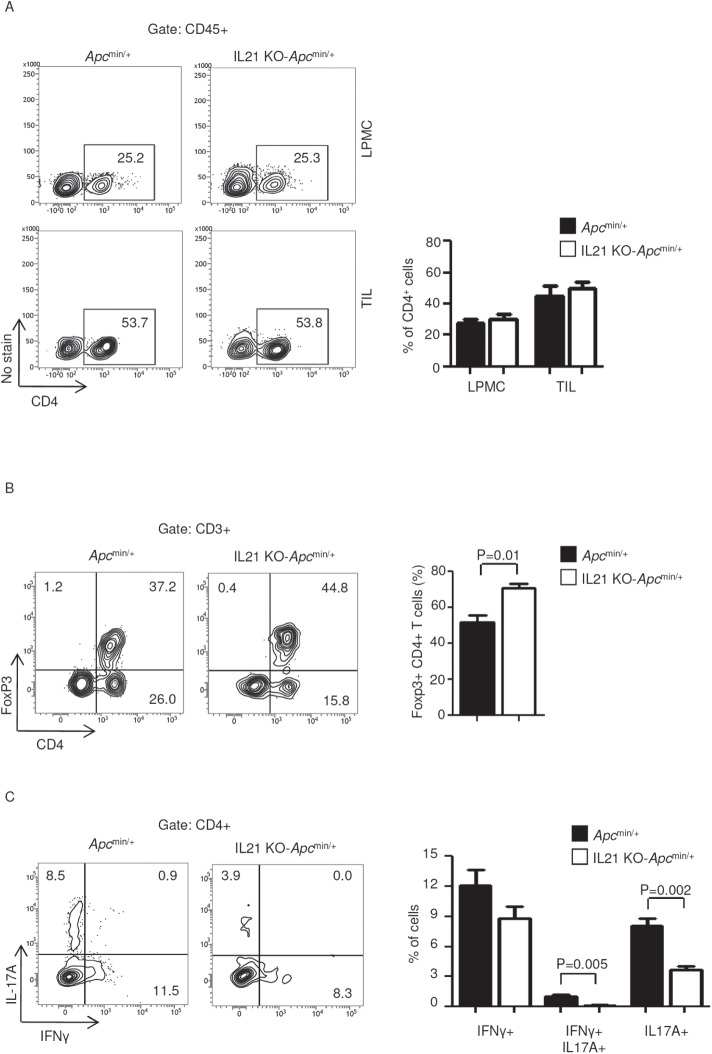
IL-21 deficiency in *Apc*^min/+^ mice associates with a functional switch of TIL-derived CD4-positive cells A. Representative dot-plots showing CD4-positive cells in LPMCs and TILs isolated from the colon of *Apc*^min/+^ mice and IL-21 KO-*Apc*^min/+^ mice killed on day 56. Numbers indicate the percentages of cells in the designated quadrants. One of four representative experiments is shown. Right insets. Representative histograms showing the fraction of CD4-positive cells in LPMCs and TILs isolated from the colon of *Apc*^min/+^ mice and IL-21 KO-*Apc*^min/+^ mice killed on day 56. CD45-positive cells were gated and analyzed for the indicated markers. Values are mean ± SEM of two independent experiments containing at least two mice per group. B. TILs isolated from the colon of *Apc*^min/+^ mice and IL-21 KO-*Apc*^min/+^ mice killed on day 56 were assessed for Foxp3-expressing CD4-positive cells by flow cytometry. The numbers indicate the percentage of cells in the designated quadrants. Graph shows the fraction of Foxp3-positive CD4 T cells in TILs isolated from the colon of *Apc*^min/+^ mice and IL-21 KO-*Apc*^min/+^ mice killed on day 56. Data are expressed as mean ± SEM of two independent experiments containing at least two mice per group. C. TILs isolated from the colon of *Apc*^min/+^ mice and IL-21 KO-*Apc*^min/+^ mice killed on day 56 were assessed for IFN-γ- and/or IL-17A-expressing CD4-positive cells by flow cytometry. The numbers indicate the percentage of cells in the designated quadrants. Graph shows the fraction of IFN-γ- and/or IL-17A-expressing CD4 T cells in TILs isolated from the colon of *Apc*^min/+^ mice and IL-21 KO-*Apc*^min/+^ mice killed on day 56. Data are expressed as mean ± SEM of two independent experiments containing at least two mice per group.

Because CD4+ T cells play a pivotal role in the growth of sporadic CRC, we next determined whether the diminished tumor incidence and severity in IL-21-deficient mice was associated with reduced infiltration and/or function of colonic CD4+ cells. Although lack of IL-21 did not alter the number of CD4-expressing LPMCs and TILs (Figure 4A), TILs isolated from IL-21-deficient *Apc*^min/+^ mice showed an increased fraction of CD4+Foxp3+ T cells (Figure 4B) and a diminished percentage of IL-17A-positive and IFN-γ/IL-17A double-positive CD4 T cells (Figure 4C).

### Reduced STAT3/NF-kB activation and reduced expression of IL-17A, IL-22, TNF-α and IL-6 in the tumors of IL-21 KO-*Apc*^min/+^ mice

**Figure 5:**
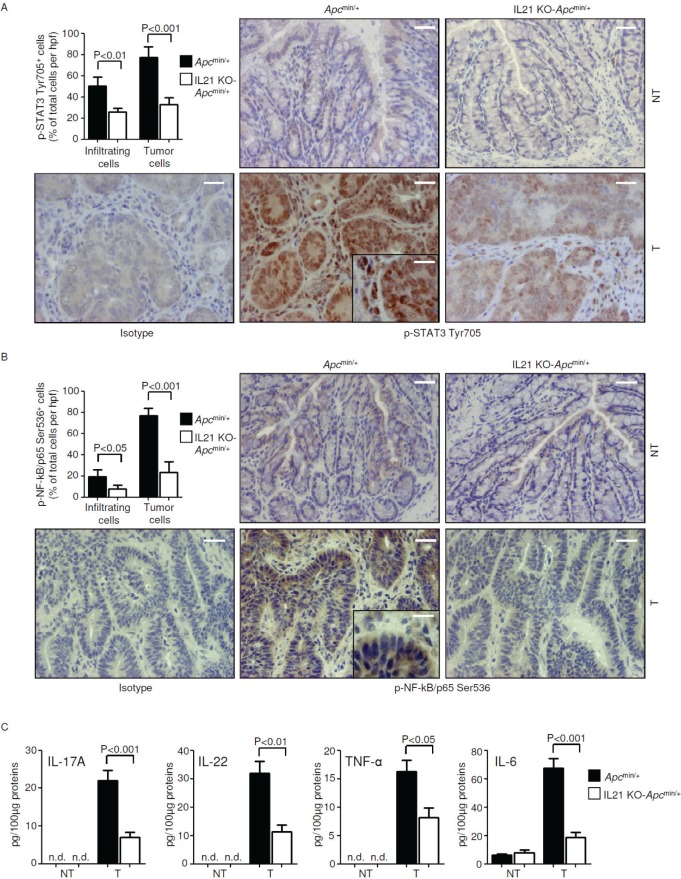
Reduced STAT3/NF-kB activation and reduced expression of IL-17A, IL-22, TNF-α and IL-6 in the tumors of IL-21 KO-*Apc*^min/+^ mice A-B. Representative images showing p-STAT3 Tyr705- (A) or p-NF-kB/p65 Ser536-positive cells (B) in colon sections taken from *Apc*^min/+^ mice and IL-21 KO-*Apc*^min/+^ mice killed on day 56. Staining with isotype control IgG is also shown. The scale bars are 20μm. The scale bar in the inset is 10μm. One of four representative experiments is shown. Upper left insets. Quantification of p-STAT3 Tyr705- (A) or p-NF-kB/p65 Ser536-positive (B) infiltrating and epithelial cells in colon sections taken from the tumor areas of *Apc*^min/+^ mice and IL-21 KO-*Apc*^min/+^ mice killed on day 56. Data are presented as mean values of positive cells per high power field (hpf) ± SEM of two independent experiments in which at least two sections per group were analyzed. NT, non-tumor area, T, tumor area. C. IL-17A, IL-22, TNF-α and IL-6 expression was assessed by ELISA in colon tissues taken from *Apc*^min/+^ mice and IL-21 KO-*Apc*^min/+^ mice killed on day 56. Data indicate mean ± SEM of two independent experiments in which at least four mice per group were considered.

Hyper-activation of the transcription factors STAT3 and NF-kB makes a major contribution to the tumorigenic process in the colon. To determine whether the reduced carcinogenesis observed in IL-21 KO-*Apc*^min/+^ mice was associated with a reduced activation of STAT3 and/or NF-kB, we compared p-STAT3 Y705 and p-NF-kB/p65 Ser536 expression in non-tumor and tumor tissues derived from colonic extracts of control and IL-21 KO-*Apc*^min/+^ mice killed on day 56. Robust activation of both STAT3 and NF-kB was seen in the neoplastic areas of control mice compared to those derived from IL-21 KO-*Apc*^min/+^ mice, whereas p-STAT3 Y705 and p-NF-kB/p65 Ser536 expression was barely detectable in non-tumor areas of both control and IL-21-deficient Apcmin/+ mice (Suppl. Figure 5A). Similar findings were seen when STAT3 and NF-kB activation was assessed by immunohistochemistry (Figure 5A and B), which also revealed a reduction of cells positive for p-STAT3 Y705 and p-NF-kB/p65 Ser536 in both transformed epithelial cells and tumor infiltrating cells in the lesions of IL-21 KO-*Apc*^min/+^ mice (Figure 5A and B).

As the ability of immune/inflammatory cells to activate protumorigenic signals in transformed cells is mostly dependent on cytokines and IL-21 regulates the induction of multiple protumorigenic cytokines [20, 21], we next analyzed the cytokine profile in the colon of control and IL-21 KO-*Apc*^min/+^ mice. The STAT3- or NF-kB-activating cytokines IL-17A, IL-22, TNF-α and IL-6 were reduced in the tumors of IL-21 KO-*Apc*^min/+^ mice at both the RNA and protein level (Figure 5C and Suppl. Figure 5B), whereas such cytokines were barely detectable in non-tumor areas of both control and IL-21 KO-*Apc*^min/+^ mice (Figure 5C and Suppl. Figure 5B).

Taken together, these results suggest that elevated levels of IL-21 in the tumors of *Apc*^min/+^ mice support a tumor-promoting inflammatory microenvironment characterized by enhanced production of IL-17A, IL-22, TNF-α and IL-6 and hyper-activation of STAT3/NF-kB.

### IL-21 indirectly affects proliferation and STAT3/NF-kB activation in mouse CRC cells

**Figure 6:**
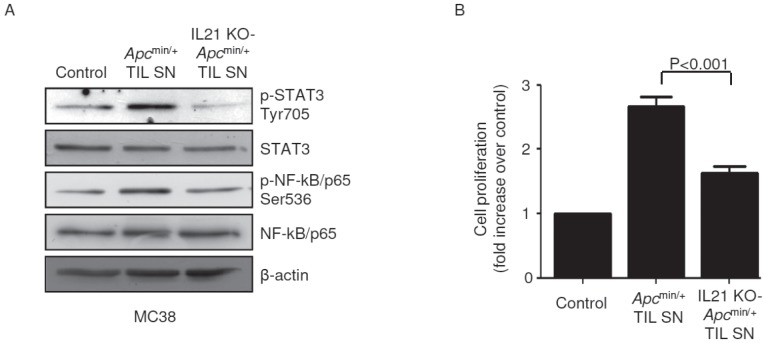
STAT3/NF-kB activation and increased proliferation are seen in mouse CRC cells cultured in the presence of TIL-derived supernatants (TIL SNs) obtained from *Apc*^min/+^ mice compared to those obtained from IL-21 KO-*Apc*^min/+^ mice A. MC38 cells were cultured in the presence of either *Apc*^min/+^ mice-derived TIL SNs or IL-21 KO-*Apc*^min/+^ mice-derived TIL SNs or RPMI 1640 complete medium (control) (all used at 1:20 final dilution) for 15 min. P-STAT3 Tyr705, STAT3, p-NF-kB/p65 Ser536 and NF-kB/p65 expression was assessed by western blotting. β-Actin was used as loading control. Shown is one of four representative experiments in which TIL SNs derived from four *Apc*^min/+^ mice and four IL-21 KO-*Apc*^min/+^ mice were used. B. MC38 cells were cultured as indicated in A. After 24 h, cell proliferation was assessed by BrdU assay. Data indicate mean ± SEM of four independent experiments.

To determine whether the pro-tumorigenic effect of IL-21 in our experimental model of CRC was due to a direct action of the cytokine on transformed epithelial cells, we used immortalized CRC cells derived from C57BL/6J mice (that is, MC38). IL-21 affected neither STAT3/NF-kB activity nor proliferation rate in MC38 cells (Suppl. Figure 6A and B). Of note, both STAT3 and NF-kB were activated in MC38 cells when cultured in the presence of TIL SNs derived from *Apc*^min/+^ mice but not from IL-21 KO-*Apc*^min/+^ mice (Figure 6A). Consistently, *Apc*^min/+^ mice-derived TIL SNs significantly increased MC38 cell growth compared with IL-21 KO-*Apc*^min/+^ mice-derived TIL SNs (Figure 6B).

### IL-21 deficiency impairs COX-2/PGE2 pathway and angiogenesis in the tumors of *Apc*^min/+^ mice

**Figure 7:**
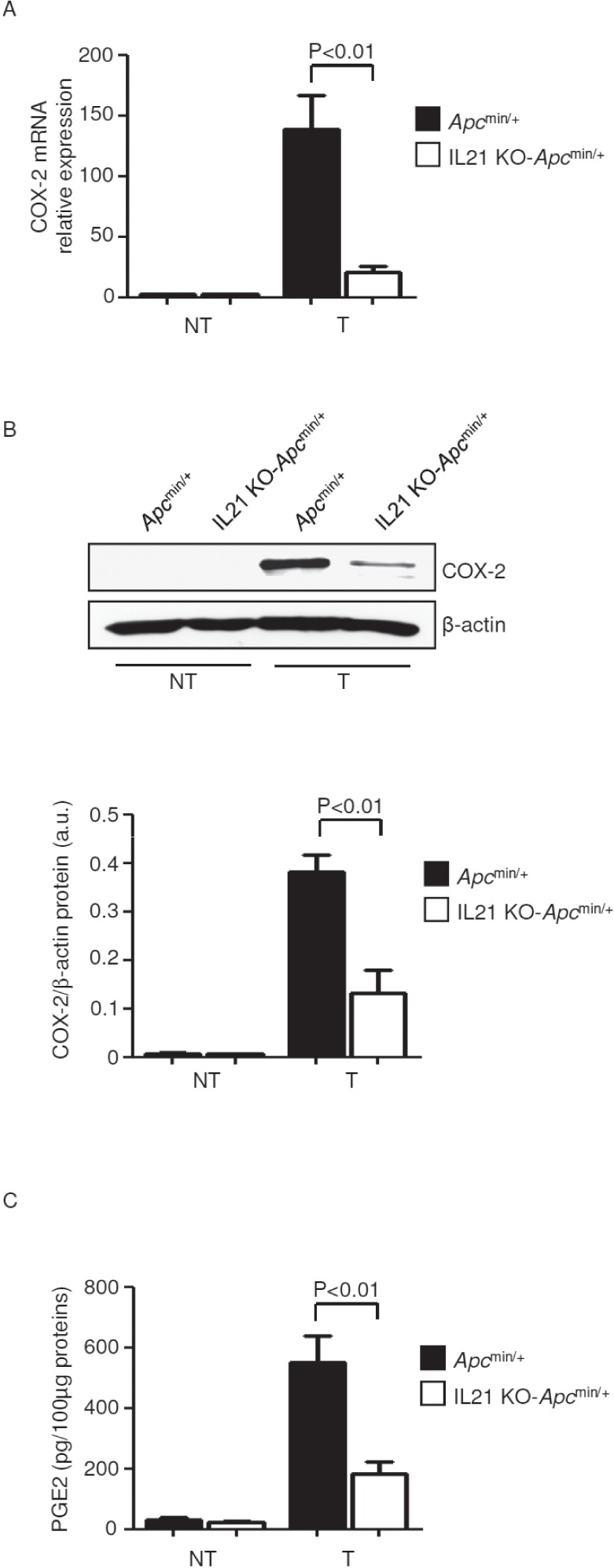
Reduced expression of COX-2/PGE2 in the tumors of IL-21 KO-*Apc*^min/+^ mice A COX-2 was assessed by real-time PCR in colon tissues taken from *Apc*^min/+^ mice and IL-21 KO-*Apc*^min/+^ mice killed on day 56. Data indicate mean ± SEM of two independent experiments in which at least two mice per group were considered. NT, non-tumor area, T, tumor area. B. Representative western blotting showing COX-2 in colon tissues taken from *Apc*^min/+^ mice and IL-21 KO *Apc*^min/+^ mice killed on day 56. β-actin was used as loading control. One of four representative experiments is shown. Bottom inset. Quantitative analysis of COX-2/β-actin protein ratio in total extracts of T and NT colon tissues taken from *Apc*^min/+^ mice and IL-21 KO-*Apc*^min/+^ mice killed on day 56 as measured by densitometry scanning of western blots. Values are expressed in arbitrary units (a.u.) and indicate the mean ± SEM of all experiments (n=4). C. PGE2 levels were assessed by ELISA in colon tissues taken from *Apc*^min/+^ mice and IL-21 KO-*Apc*^min/+^ mice killed on day 56. Data indicate mean ± SEM of two independent experiments in which at least three mice per group were considered.

Since induction of COX-2 and its product PGE2 by IL-6 and TNF-α positively influences colorectal tumorigenesis [22], we next examined whether IL-21 deficiency affects the COX-2/PGE2 axis in *Apc*^min/+^ mice. In both control and IL-21 KO-*Apc*^min/+^ mice, COX-2 RNA and protein were increased in the tumors as compared to non-tumor areas (Figure 7A and B), though induction of COX-2 was markedly diminished in the absence of IL-21 (Figure 7A and B). Consistently, lack of IL-21 markedly decreased PGE2 in the tumors of *Apc*^min/+^ mice (Figure 7C). Our results are in line with previous reports showing that deletion of the Cox-2 gene in *Apc*^min/+^ mice results in a significant reduction in both the number and size of lesions [23, 24].

**Figure 8:**
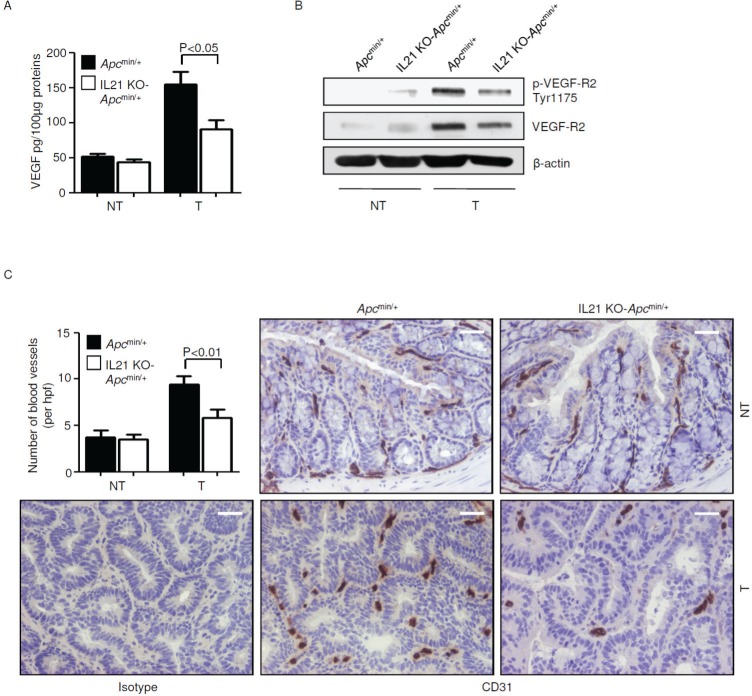
Reduced angiogenesis in the tumors of IL-21 KO-*Apc*^min/+^ mice A. VEGF expression was assessed by ELISA in colon tissues taken from *Apc*^min/+^ mice and IL-21 KO-*Apc*^min/+^ mice killed on day 56. Data indicate mean ± SEM of two independent experiments in which at least four mice per group were considered. NT, non-tumor area, T, tumor area. B. Representative western blotting showing p-VEGF-R2 Tyr1175 and VEGF-R2 expression in colon tissues taken from *Apc*^min/+^ mice and IL-21 KO-*Apc*^min/+^ mice killed on day 56. β-actin was used as loading control. One of four representative experiments in which similar results were obtained is shown. C. Representative immunohistochemical staining of CD31 in colon sections taken from *Apc*^min/+^ mice and IL-21 KO-*Apc*^min/+^ mice killed on day 56. Staining with isotype control IgG is also shown. The scale bars are 20μm. One of four representative experiments is shown. Upper left inset. Quantification of blood vessels in colon sections taken from *Apc*^min/+^ mice and IL-21 KO-*Apc*^min/+^ mice killed on day 56. Data are presented as mean values of blood vessels per high power field (hpf) ± SEM of two independent experiments in which at least two sections per group were analyzed.

VEGF signaling has been identified as a key factor in the pathogenesis of sporadic colorectal tumors in mouse and man [25, 26], and both PGE2 and IL-17A are known to induce VEGF [27, 28]. Therefore, we investigated whether IL-21 controls VEGF and VEGF-related angiogenic pathways in the *Apc*^min/+^ mouse model. Knock-down of IL-21 reduced VEGF, p-VEGF-R2 Tyr1175 and VEGF-R2 in the tumors of *Apc*^min/+^ mice (Figure 8A and B). Consistently, a significant decrease in CD31-positive blood vessels, reflective of a diminished neo-angiogenesis, was seen in the tumors of IL-21 KO-*Apc*^min/+^ mice compared with control tumors (Figure 8C). No significant change in VEGF, p-VEGF-R2 Tyr1175 and VEGF-R2 expression as well as in the number of blood vessels was seen in the normal colon between control and IL-21 KO-*Apc*^min/+^ mice (Figure 8A-C).

## DISCUSSION

This study was undertaken to clarify the role of IL-21 in sporadic CRC. Our data confirm and expand on our previous work showing up-regulation of IL-21 in the tumor areas of patients with sporadic CRC. We here also show that CD3+CD8− cells infiltrating the tumor tissue are the major source of IL-21.

Within the tumor microenvironment, expression of Th1-associated molecules positively correlates with reduced recurrence of sporadic CRC [29], whereas accumulation of Th17 cells impacts negatively on the prognosis of patients with such a neoplasia [17, 18]. We investigated the phenotypic characteristic of IL-21-producing CD3+CD8− cells by flow cytometry and determined whether IL-21 was co-expressed with Th1 and/or Th17-related transcription factors (that is, T-bet and RORγt respectively) and cytokines (that is, IFN-γ and IL-17A respectively). Our data indicate that IL-21 is preferentially made by T-bet/RORγt double-positive Th cells and, to a lesser extent, by T-bet-RORγt+ Th cells. More than 50% of IL-21 producing Th cells co-express IFN-γ, thus confirming our previous studies showing that IFN-γ-expressing cells are a major source of IL-21 in the colon of patients with inflammatory bowel disease [30]. Foxp3+RORγt+ T regulatory cells (Tregs) expand in CRC patients and may contribute to tumor development in part through the synthesis of IL-17A [31]. However, flow cytometry analysis of TILs showed that T-bet/RORγt double-positive or T-bet-RORγt+ Foxp3-expressing cells did not produce IL-21. This is consistent with the demonstration that Foxp3 binds to *IL-21* gene promoter and suppresses IL-21 production in CD4+ cells [32].

STAT3 and NF-kB are involved in the interplay between immune/inflammatory and malignant cells and activation of such transcription factors positively modulates CRC cell proliferation and survival [19]. This notion, together with previous evidence showing that gut epithelial cells express IL-21R and respond to IL-21 [33], prompted us to investigate whether IL-21 can activate STAT3 and NF-kB in human CRC cells. IL-21 failed to activate both factors in DLD-1 and HT-29 cells. Moreover, IL-21 did not affect proliferation and survival of such cells, thus arguing against a role for this cytokine in directly modulating malignant cell growth in colon carcinogenesis. In contrast, addition of a neutralizing IL-21 antibody to cultures of TILs isolated from patients with sporadic CRC and maximally activated with anti-CD3/CD28 reduced IL-17A and IL-22 production. This finding well fits with both *in vitro* and *in vivo* studies showing a key role of IL-21 in sustaining Th17 immune responses in the gut [21, 34].

IL-21 was upregulated in the colon lesions of *Apc*^min/+^ mice as compared with the non-tumor mucosa and lack of IL-21 in such mice resulted in a dramatic reduction of both tumor incidence and size. In both LPMCs and TILs, IL-21 deficiency did not significantly alter the fraction of cytotoxic lymphocytes and NK cells and resulted in a diminished production of cytotoxic molecules by such cell populations in TILs. These data are consistent with previous studies showing that IL-21 is important for activity of CD8 and NK cells [11] and indicate that resistance of IL-21 KO-*Apc*^min/+^ mice against colon tumorigenesis is not due to enhanced immune surveillance. Similarly, no change in the percentage of bulk population of CD4+ cells was seen in LPMCs and TILs isolated from control and IL-21-deficient *Apc*^min/+^ mice. Further analysis of CD4+ T cell subsets in TILs showed that IL-21 deficiency increased the fraction of Foxp3+ CD4+ T cells. These data are not surprising as we previously reported a similar effect for IL-21 in a mouse model of colitis-associated CRC [9] and are in line with the notion that IL-21 suppresses the peripheral differentiation of Tregs [36]. In this context it is noteworthy that, although the role of Tregs in CRC is controversial and still under debate [37], regulatory T lymphocytes can induce regression of intestinal tumors in *Apc*^min/+^ mice [38]. In line with human data, lack of IL-21 associated with a diminished percentage of IL-17A-producing CD4+ T cells in TILs. Overall the above findings indicate that IL-21 can affect the function of CD4+ T cells in TILs.

The tumor tissues of IL-21 KO-*Apc*^min/+^ mice exhibited reduced activation of STAT3/NF-kB and this was evident in both epithelial and immune cells. Mouse tumor epithelial cells do not express IL-21R [9], and stimulation of immortalized CRC cells derived from C57BL/6J mice with IL-21 does not affect cell growth and activation of STAT3/NF-kB. Altogether these data suggest that the reduced activation of STAT3/NF-kB observed in cancer cells of IL-21-deficient *Apc*^min/+^ mice is not due to the lack of a direct effect of IL-21 on these cells but probably reflects the diminished synthesis of IL-17A, IL-22, TNF-α and IL-6, given that these cytokines directly activate STAT3 (IL-22 and IL-6) or NF-kB (IL-17A and TNF-α) in malignant cells [19]. Since activation of STAT3 and NF-kB in immune cells enhances the production of Th17-related cytokines, including IL-21 itself [21, 39, 40], it is conceivable that IL-21 could be part of a positive feedback loop that amplifies tumorigenic signals in the colon. In support of this hypothesis, *in vitro* stimulation of mouse CRC cells with TIL SNs derived from *Apc*^min/+^ mice, but not from IL-21 KO *Apc*^min/+^ mice, promotes STAT3/NF-kB activation and cell proliferation.

Th17-related cytokines control induction of other pathways implicated in the process of colon tumorigenesis (for example, COX-2, VEGF). Therefore, it is not surprising that the reduced tumorigenesis documented in the absence of IL-21 was associated with reduced activation of COX-2/PGE2 pathway and diminished neo-angiogenesis. These findings do not however exclude the possibility that the reduced expression of COX-2 and VEGF in IL-21 KO-*Apc*^min/+^ mice may be linked to the effect of IL-21 on additional cells other than Th17. Indeed, it has previously been reported that IL-21 can target and modulate the function of endothelial cells in other systems [41]. Importantly, the lack of IL-21 would seem to affect tumor-derived but not normal angiogenesis.

In conclusion, our studies reveal for the first time the crucial involvement of IL-21 in the mechanisms that control the growth of sporadic CRC.

## MATERIALS AND METHODS

### Patients and samples

Paired tissue samples were taken from the tumor area and the macroscopically unaffected, adjacent, colonic mucosa of 10 patients who underwent colon resection for sporadic CRC (all with TNM stage II-III) at the Tor Vergata University Hospital (Rome, Italy). These samples were used to extract total proteins as described elsewhere [42]. TILs were isolated from tumor samples as described below. No patients received radiotherapy or chemotherapy prior to undergoing surgery. The human studies were approved by the local ethics committee and each patient gave written informed consent.

### Animals

*Apc*^min/+^ mice, which spontaneously develop intestinal tumors, were obtained from Jackson (Bar Harbor, ME). IL-21 KO (129S5-Il21tm1Lex) mice were purchased from Lexicon Genetics Incorporated (The Woodlands, TX). IL-21 KO mice are viable and do not exhibit any phenotype. Both *Apc*^min/+^ mice and IL-21 KO mice were established on a C57BL/6J background. Male *Apc*^min/+^ mice were crossed with female IL-21 KO mice to obtain IL-21 KO-*Apc*^min/+^ mice. Mice were routinely tested for health status and infections according to the FELASA guidelines. Mice were negative for all pathogens included in this protocol. Mice were also negative for *Helicobacter hepaticus* and *Helicobacter bilis*. All animal experiments were approved by the local Institutional Animal Care and Use Committee.

### Isolation of tumor infiltrating leukocytes from human colonic samples

Tumor infiltrating leukocytes were isolated from CRC samples using dithiothreitol–ethylenediaminetetraacetic acid and collagenase method as previously described [43].

### Cell cultures

All reagents were from Sigma-Aldrich (Milan, Italy) unless otherwise specified. The human-derived, nontransformed colonic epithelial cell line NCM460 was purchased from INCELL Corporation (San Antonio, TX) and maintained in M3:10 medium (INCELL Corporation). The human CRC cell lines DLD-1 and HT-29 were obtained from the American Type Culture Collection (ATCC, Manassas, VA) and cultured in RPMI 1640 and McCoy's 5A medium respectively. All media were supplemented with 10% fetal bovine serum, 1% penicillin/streptomycin (both from Lonza, Verviers, Belgium) and 50μg/ml gentamycin. The murine CRC cell line MC38 was generously provided by Dr. Ignacio Melero (Universidad de Navarra, Pamplona, Spain) and maintained in DMEM medium supplemented with 10% fetal bovine serum and 1% penicillin/streptomycin.

Cells were maintained in a 37°C, 5% CO2, fully humidified incubator. Cell lines have been recently authenticated by STR DNA fingerprinting using the PowerPlex 18D System kit according to the manufacturer's instructions (Promega, Milan, Italy). The STR profiles of all the cell lines matched the known DNA fingerprints.

To characterize IL-21 producing cells, freshly isolated TILs were cultured in the presence of phorbol 12-myristate 13-acetate (PMA, 80 pM), ionomycin (1 mg/mL), and monensin (2 μM, eBioscience, San Diego, CA) for 5 h, and then evaluated by flow cytometry following staining with specific fluorochrome-conjugated antibodies. An aliquot of cells was resuspended and cultured in complete RPMI 1640 medium and cell-free supernatants harvested after 48 h. To assess whether IL-21 activates STAT3 and/or NF-kB in human CRC cells, DLD-1 and HT-29 cells were either left untreated or cultured in the presence of either IL-21 (25-100 ng/ml, Life Technologies, Milan, Italy) or TIL-derived supernatants (used at 1:20 final dilution) for 15 min.

To determine whether IL-21 activates STAT3 and/or NF-kB in mouse CRC cells, MC38 cells were either left untreated or cultured in the presence of either IL-21 (25-100 ng/ml, R&D Systems, Milan, Italy) or TIL SNs derived from *Apc*^min/+^ mice (used at 1:20 final dilution) for 15 min. To examine whether IL-21 affects human CRC cell proliferation and/or viability, DLD-1 and HT-29 cells were eiher left untreated or cultured in the presence of either IL-21 (25-100 ng/ml) or TIL-derived supernatants (used at 1:20 final dilution) or staurosporine for 12-72 h. To examine whether IL-21 affects MC38 cell proliferation, cells were eiher left untreated or cultured in the presence of either IL-21 (25-100 ng/ml) or TIL SNs derived from *Apc*^min/+^ mice (used at 1:20 final dilution) for 24 hours.

To determine whether IL-21 controls pro-tumorigenic signals in human TILs, cells were isolated from sporadic CRC of 4 patients, activated with anti-CD3/CD28 in the presence of either a specific antibody neutralizing IL-21 (anti-IL-21, used at 10 μg/ml, Giuliani S.p.A., Milan, Italy) or control IgG (R&D Systems, Milan, Italy) and examined by flow cytometry after 24 h.

### Assessment of cell proliferation and death

Cell proliferation was assessed by using a commercially available 5-bromodeoxyuridine (BrdU) assay kit (Roche Diagnostic). To assess cell death and apoptosis, cells were stained with fluorescein isothiocyanate-conjugated annexin V (AV, Immunotools, Friesoythe, Germany) and 5μg/ml propidium iodide (PI) for 30 min at 4°C, and their fluorescence measured by flow cytometry.

### Western blotting

Total proteins were extracted from human CRC cells or human and mouse colonic tissues as described elsewhere [42] and separated by SDS/PAGE. Blots were incubated with antibodies to IL-21 (Millipore, Milan, Italy), p-STAT3 Tyr705, p-NF-κB/p65 Ser536, p-VEGF-R2 Tyr1175, VEGF-R2 (all from Cell Signaling, Danvers, MA), STAT3, p65 and IL-21R antibodies (all from Santa Cruz Biotechnology, Santa Cruz, CA). To ascertain equivalent loading of the lanes, blots were stripped and incubated with an anti-β-actin antibody.

### Flow cytometry analysis

Flow cytometry analysis was performed by using a BD FACSVerse (BD Biosciences). Human TILs were stained with the following monoclonal antibodies: CD45-APC-H7, CD3ε-PerCP, CD8α-V500, CD56-PE-Cy7, CD19-FITC, IFN-γ-FITC, IL-17A-V450 (all from BD Pharmingen, Milan, Italy), IL-21-PE, T-bet-PE-Cy7 (clone 4B10), RORγt-APC (clone AFKJS-9), Foxp3-FITC (clone PCH101), IL-22-APC, IL-6 PerCP (both from eBioscience), CD68-APC (Biolegend, San Diego, CA). Mouse LPMCs and TILs were stained with the following monoclonal antibodies: CD45-APC-Cy7, CD4-PerCP, CD8α-FITC, CD49b-PE (clone DX5), FoxP3-APC, IL-17A-PE, IFN-γ-PE-Cy7 (both from BD Pharmingen), perforin-APC, granzyme B-PE-Cy7 (all from eBioscience). In parallel, cells were stained with the respective control isotype antibodies.

### Experimental model of sporadic CRC

Cohoused 6- to 7-week-old female *Apc*^min/+^ and IL-21 KO-*Apc*^min/+^ mice received intraperitoneal injections of 10 mg/kg azoxymethane (AOM) once a week for 2 weeks in order to increase colon tumorigenesis as previously reported [44]. Mice were killed 8 weeks after the first AOM injection (day 56). Colonoscopy was performed in a blinded manner for monitoring of tumorigenesis using the Coloview high-resolution mouse endoscopic system (Karl-Storz; Tuttlingen, Germany). Tumors observed during endoscopy were counted to obtain the overall number of lesions. Tumor sizes of all tumors in a given mouse were scored using the protocol described by Becker et al [45].

### Isolation of LPMCs and TILs from mouse colon tissues

LPMCs and TILs were isolated from colon non-tumor and tumor areas of Apcmin/+ mice and IL-21 KO-*Apc*^min/+^ mice as previousy described [9].

### Immunohistochemistry

Colonic cryosections of *Apc*^min/+^ mice and IL-21 KO-*Apc*^min/+^ mice were stained with p-STAT3 Tyr705, p-NF-κB/p65 Ser536 (both from Cell Signaling) and CD31 (BD Pharmingen). Isotype control-stained sections were prepared under identical conditions replacing the primary antibody with either a rabbit or rat IgG control antibody (R&D Systems). Proliferating cells were evaluated using a PCNA staining kit (ZYMED Laboratories, Carlsbad, CA) according to the manufacturer's instructions. Negative control-stained sections were prepared under identical immunohistochemical conditions omitting the primary antibody.

### Analysis of cytokine and angiogenesis-related factors in mouse colonic tissues

One hundred μg protein was assessed by enzyme-linked immunosorbent assay (ELISA) for IL-17A, IL-22, IL-6, tumor necrosis factor (TNF)-α, vascular epithelial growth factor A (VEGF-A; hereafter referred to as VEGF) (all from R&D Systems) and prostaglandin (PG)E2 (Cayman Chemical, Ann Arbor, MI) according to the manufacturer's protocols.

### RNA extraction, cDNA preparation and real-time PCR

Total RNA was extracted from tumor and non-tumor tissue of *Apc*^min/+^ mice and IL-21 KO-*Apc*^min/+^ mice using TRIzol reagent according to the manufacturer's instructions (Life Technologies). Primers were as follows: IL-17A sense: 5′-TCAGACTACCTCAACCGTTC-3′ and anti-sense 5′-TTCAGGACCAGGATCTCTTG-3′, TNF-α sense 5′-ACCCTCACACTCAGATCATC-3′ and anti-sense: 5′-GAGTAGACAAGGTACAACCC-3′, IL-6 sense: 5′-AGCCAGAGTCCTTCAGAGAG-3′ and anti-sense 5′-GATGGTCTTGGTCCTTAGCC-3′, COX-2 sense 5′-TTCTTTGCCCAGCACTTCAC-3′ and anti-sense 5′-GGATACACCTCTCCACCAAT-3′. IL-22 RNA expression was evaluated using a TaqMan assay (Life Technologies). RNA expression was calculated relative to the housekeeping β-actin gene on the base of the ΔΔCt algorithm.

### Statistical analysis

Values are expressed as mean ± SEM and results analyzed using the two-tailed Student t test. Significance was defined as P-values < 0.05.

## SUPPLEMENTARY MATERIAL AND FIGURES


