# Supplementary material for: Interleukin-21 sustains inflammatory signals that contribute to sporadic colon tumorigenesis
Source: Oncotarget. 2015 Mar 12;6(12):9908–23. doi: 10.18632/oncotarget.3532 (PMC4496406; doi:10.18632/oncotarget.3532)
Supplement: Supplementary file 1 [file oncotarget-06-9908-s001.pdf]

Interleukin-21 sustains inflammatory signals that contribute to sporadic colon tumorigenesis

Supplementary Material

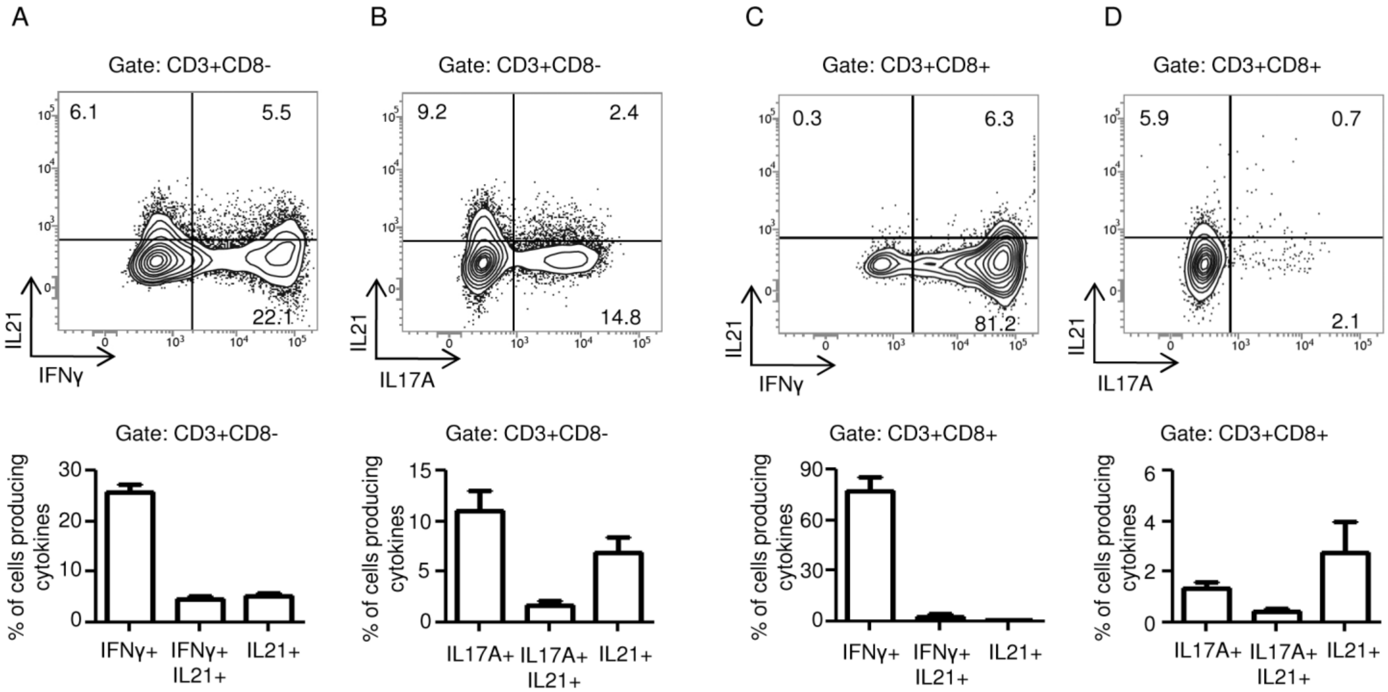

Supplementary figure 1: **A and B**. Representative dot-plots showing the fraction of IL-21+ and/or IFN-γ+ (**A**) and IL-21+ and/or IL17A+ (**B**) CD3+CD8- cells in TILs. The numbers indicate the percentage of cells in the designated quadrants. The example is representative of four experiments in which cells isolated from four patients with sporadic CRC were analyzed. Bottom panels. Representative histograms showing the fraction of IL-21+ and/or IFN-γ+ (**A**) and IL-21+ and/or IL17A+ (**B**) CD3+CD8- cells in TILs. Data are expressed as mean ± SEM of four experiments in which cells isolated from four patients with sporadic CRC were analyzed. **C and D**. Representative dot-plots showing the fraction of IL-21+ and/or IFN-γ+ (**C**) and IL-21+ and/or IL17A+ (**D**) CD3+CD8+ cells in TILs. The numbers indicate the percentage of cells in the designated quadrants. The example is representative of four experiments in which cells isolated from four patients with sporadic CRC were analyzed. Bottom panels. Representative histograms showing the fraction of IL-21+ and/or IFN-γ+ (**C**) and IL-21+ and/or IL17A+ (**D**) CD3+CD8+ cells in TILs. Data are expressed as mean ± SEM of four experiments in which cells isolated from four patients with sporadic CRC were analyzed.

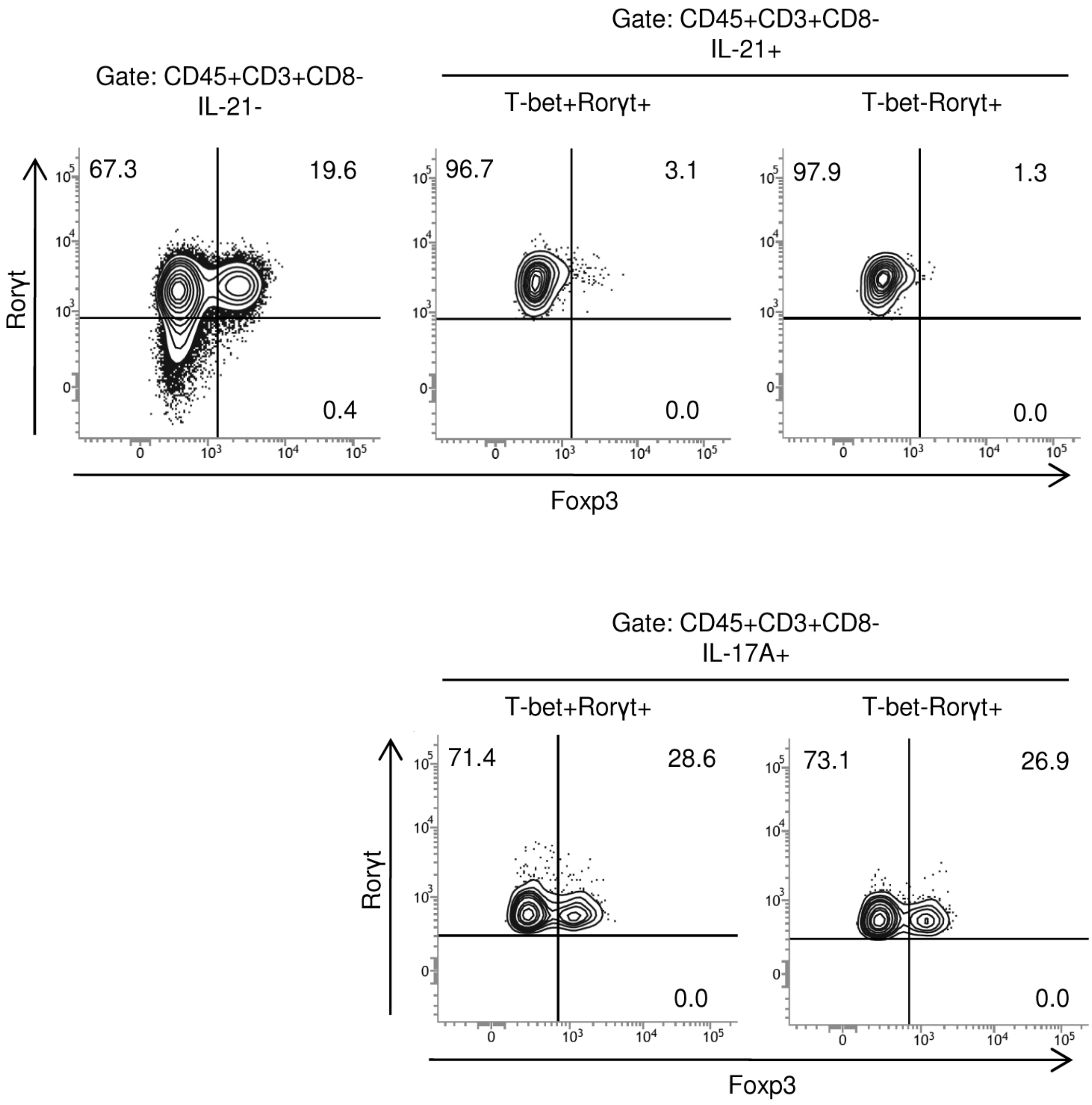

Supplementary figure 2: Co-expression of the regulatory T cell-associated transcription factor Forkhead box P3 (FoxP3) in T-bet/RORγt double-positive and T-bet-RORγt+ CD3+CD8-cells associated with IL-17A but not IL-21 production. TILs were gated as illustrated and then analyzed for the indicated markers. The numbers indicate the percentage of cells in the designated quadrants. The dot-plots are representative of four experiments in which cells isolated from four patients with sporadic CRC were analyzed.

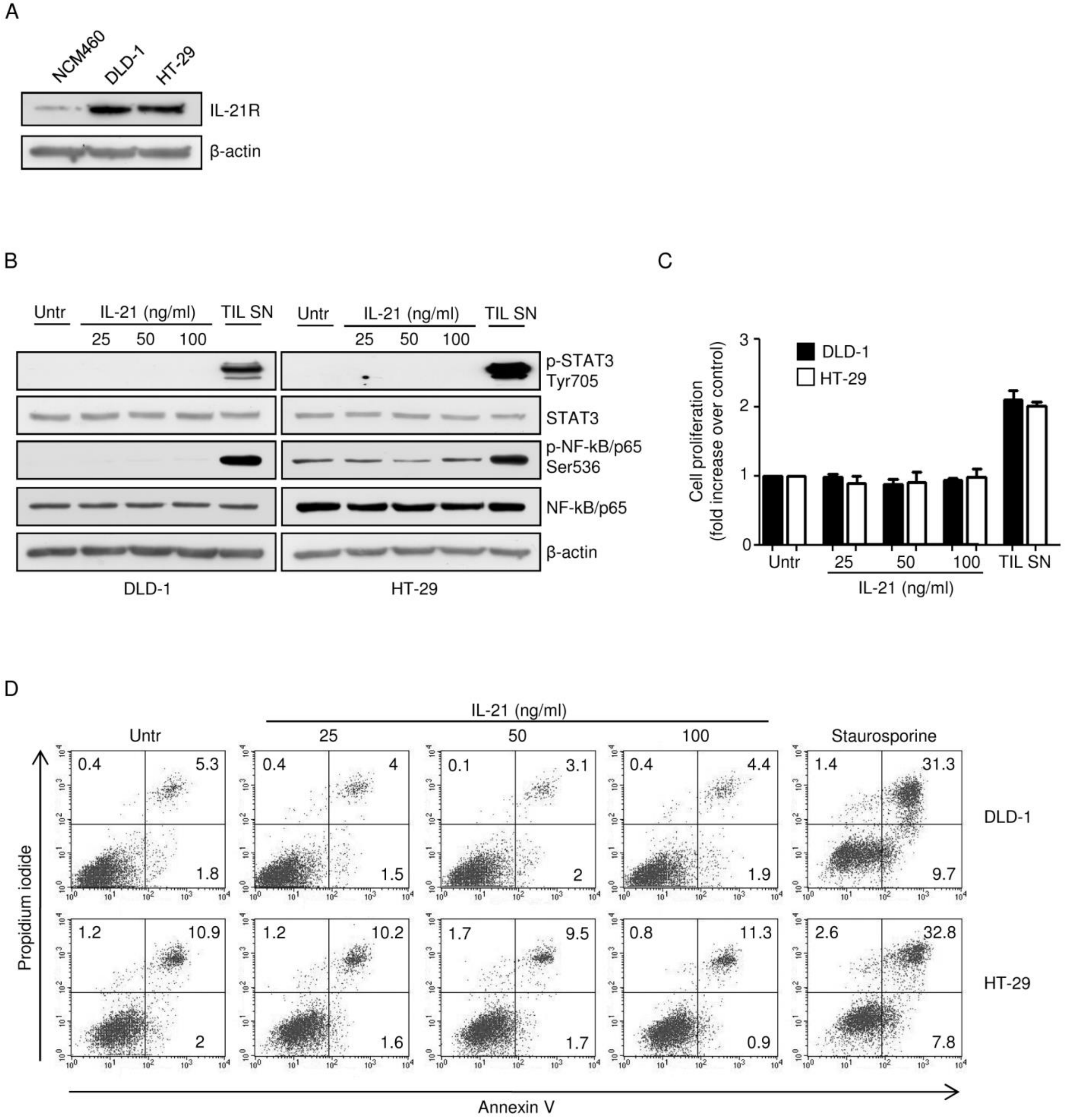

Supplementary figure 3: IL-21 does not directly affect STAT3/NF-kB activation, proliferation and survival in CRC cells. **A**. Representative western blotting showing IL-21R expression in a human-derived nontransformed colonic epithelial cell line (NCM460) and in human CRC cell lines (DLD-1 and HT-29). β-actin was used as loading control. One of two representative experiments in which similar results were obtained is shown. **B**. IL-21 fails to activate STAT3 and NF-kB in CRC cells. Representative western blotting showing p- STAT3 Tyr705, STAT3, p-NF-kB/p65 Ser536 and NF-kB/p65 expression in DLD-1 and HT-29 cells either left untreated (Untr) or stimulated with IL-21 (25-100 ng/ml) for 15 min. Tumor infiltrating leukocyte-derived supernatants (TIL SN; used at 1:20 final dilution) were used as positive control for STAT3 and NF-kB activation. β-actin was used as loading control. One of three representative experiments in which similar results were obtained is shown. **C**. IL-21 does not affect CRC cell proliferation. DLD-1 and HT-29 cells were cultured in the presence or absence of IL-21 (25-100 ng/ml) and TIL SN (used at 1:20 final dilution). After 24 h, cell proliferation was assessed by BrdU assay. Data indicate mean ± SEM of three independent experiments. **D**. Representative dot-plots showing the

percentages of Annexin V (AV)- and/or propidium iodide (PI)-positive DLD-1 cells stimulated or not with increasing doses of IL-21 (25-100 ng/ml) for 72 h. Staurosporine (1µM for 12 h) was used as a positive control for cell death induction. Numbers indicate the percentage of AV and/or PI-positive cells within the designated quadrants. One of three representative experiments in which similar results were obtained is shown.

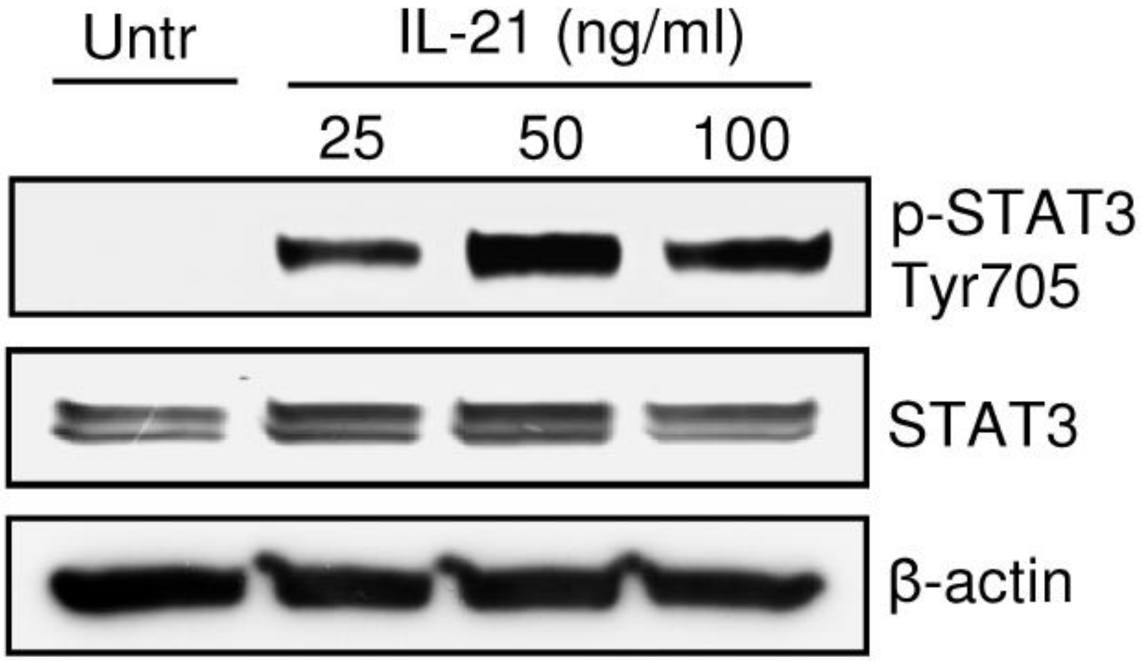

Supplementary figure 4: IL-21 activates STAT3 in human lamina propria mononuclear cells (LPMCs). LPMCs isolated from the macroscopically healthy colon mucosa of one patient who had undergone resection for sporadic CRC were either left untreated (Untr) or stimulated with the indicated doses of IL-21 for 15 min. P-STAT3 Tyr705 and STAT3 expression was assessed by western blotting. β-actin was used as loading control. One of three representative experiments in which similar results were obtained is shown.

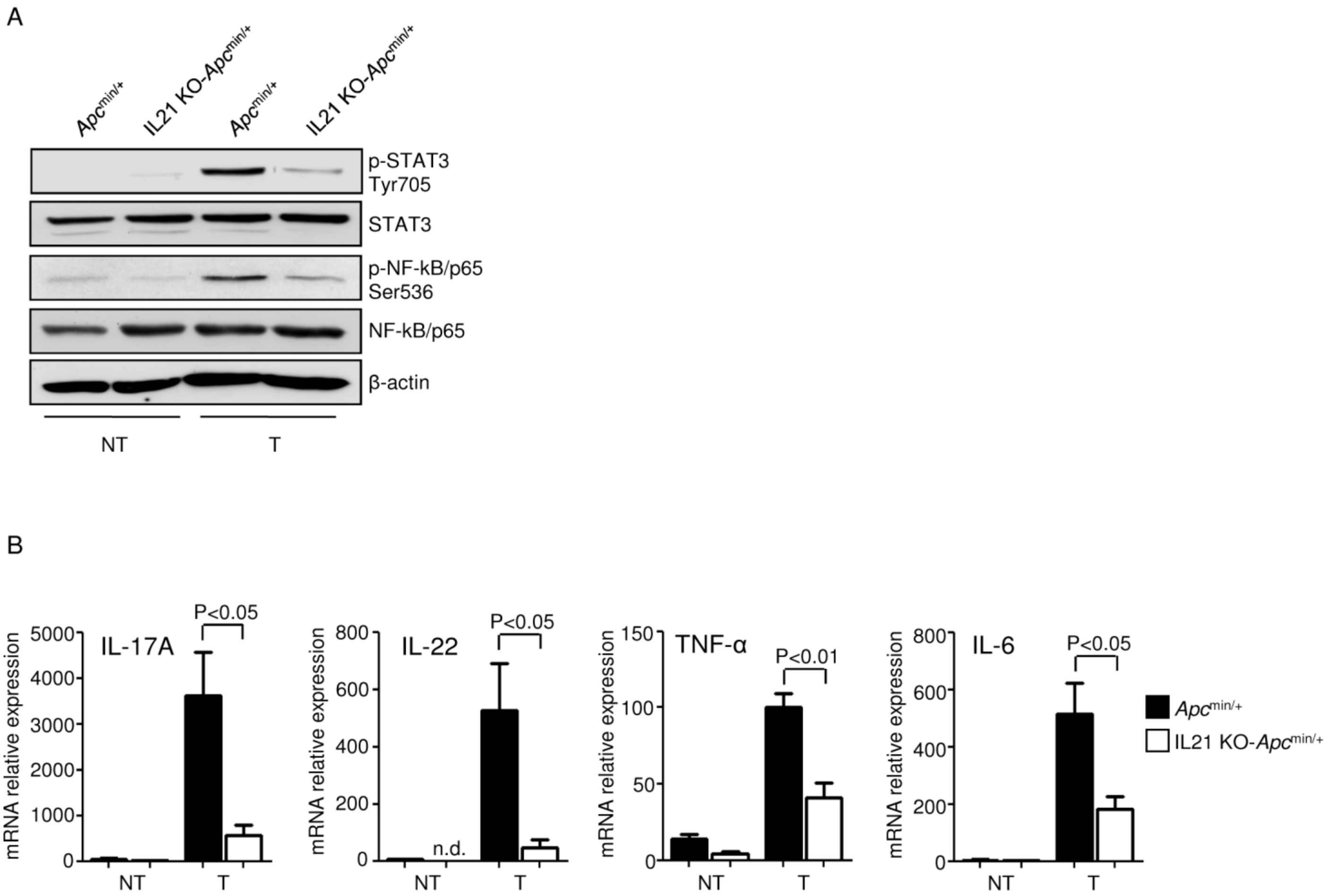

Supplementary figure 5: Reduced STAT3/NF-kB activation and reduced expression of IL-17A, IL-22, TNF-α and IL-6 in the tumors of IL-21 KO-Apc$^{min/+}$ mice. **A**. Representative western blotting showing p-STAT3 Tyr705, STAT3, p-NF-kB/p65 Ser536 and NF-kB/p65 expression in colon tissues taken from Apc$^{min/+}$ mice and IL-21 KO-Apc$^{min/+}$ mice killed on day 56. β-actin was used as loading control. One of three representative experiments in which similar results were obtained is shown. NT, non-tumor area, T, tumor area. **B**. IL-17A, IL-22, TNF-α and IL-6 expression was assessed by ELISA in colon tissues taken from Apc$^{min/+}$ mice and IL-21 KO-Apc$^{min/+}$ mice killed on day 56. Data indicate mean ± SEM of two independent experiments in which at least two mice per group were considered. Differences were calculated using the two-tailed Student t-test. NT, non-tumor area, T, tumor area, n.d., not detectable.

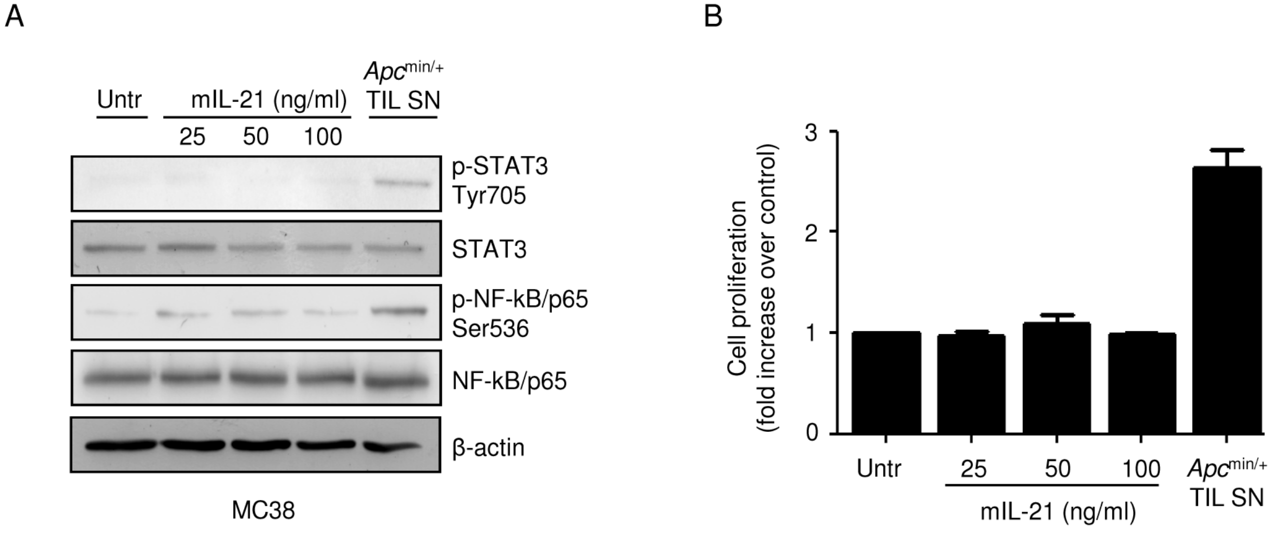

Supplementary figure 6: IL-21 fails to activate STAT3 and NF-kB and to increase proliferation rate in mouse CRC cells. **A**. Representative western blotting showing p-STAT3 Tyr705, STAT3, p-NF-kB/p65 Ser536 and NF-kB/p65 expression in MC38 cells either left untreated (Untr) or stimulated with IL-21 (25-100 ng/ml) for 15 min. Apc$^{min/+}$ mice-derived TIL SNs (used at 1:20 final dilution) were used as positive control for STAT3 and NF-kB activation. β-actin was used as loading control. One of three representative experiments in which similar results were obtained is shown. **B**. IL-21 does not affect mouse CRC cell proliferation. MC38 cells were cultured in the presence or absence of IL-21 (25-100 ng/ml). Apc$^{min/+}$ mice-derived TIL SNs (used at 1:20 final dilution) were used as positive control. After 24 h, cell proliferation was assessed by BrdU assay. Data indicate mean ± SEM of three independent experiments.
